# Biliary atresia in a neonate with a history of COVID-19: A case report

**DOI:** 10.1016/j.ijscr.2021.106705

**Published:** 2021-12-20

**Authors:** Steven W. Thornton, Raluca E. Gosman, Debra L. Sudan, Henry E. Rice, Mitchell K. Arbogast, Tamara N. Fitzgerald

**Affiliations:** a Duke University School of Medicine, Durham, NC, USA; b Department of Pathology, Duke University Medical Center, NC, USA; c Department of Surgery, Duke University Medical Center, Durham, NC, USA

**Keywords:** Biliary atresia, Kasai, Hepatic, Biliary, Case report, COVID-19

## Abstract

**Introduction and importance:**

Biliary Atresia is the progressive destruction of the neonatal intra- and extra- hepatic bile ducts. The novel coronavirus has shown dramatic hepatic tropism, and patients experiencing liver injury appear to have worse outcomes. We present the first documented case of a neonate diagnosed with Biliary Atresia and a prior history of COVID-19.

**Case presentation:**

A two-month-old female presented with increasing scleral icterus. Her laboratory testing demonstrated direct hyperbilirubinemia, with elevated alkaline phosphatase and increased ALT. She tested positive for COVID-19 at that time, requiring a two-week quarantine during which time she did not develop respiratory symptoms. Two weeks later, she presented to the hospital with emesis and an evaluation concerning for biliary atresia. She ultimately underwent a Kasai repair and recovered well with no significant post-operative complications.

**Clinical discussion:**

Biliary Atresia is a heterogenous disease of unknown etiology, though viral triggers are suggested to contribute. COVID-19 disease is frequently associated with liver damage, though its relationship to Biliary Atresia is unexplored. We present a case of a neonate who contracted COVID-19 infection, and subsequently developed biliary atresia.

**Conclusion:**

Considering this child's concurrent COVID-19 infection, viral mediated hepatic and biliary inflammation may have contributed to the development of Biliary Atresia in this case. The proposed relationship requires additional investigation but may suggest value in COVID-19 testing for patients presenting with Biliary Atresia.

## Highlights

• Biliary atresia results in a fibrosclerosing, obliterative cholangiopathy
• Several viruses may play a role in the development of biliary atresia
• The novel coronavirus *SARS-CoV-2* has shown dramatic hepatic tropism
• It is unknown if COVID-19 disease is related to the development of biliary atresia
• We present the first case of biliary atresia after diagnosis of COVID-19 disease

## 1 Introduction

Biliary atresia is the progressive destruction of intra- and extra-hepatic bile ducts in neonates, leading to an obstructive cholangiopathy [1], [2]. Although the etiology remains unclear, there are several potential causative factors, all ending in obliterative cholangiopathy [1]. Theories for the pathogenesis of biliary atresia include *de novo* primary immunologic injury, genetic predispositions, and secondary immunologic injury. Many viral candidates have been studied, with inconclusive evidence to confirm a causal relationship [3]. With the discovery of a novel coronavirus in 2019 (COVID-19), the relationship between COVID-19 and biliary atresia is unknown.

Features of COVID-19 are well described, but the mechanism of liver injury is not clearly understood [4]. Additionally, the clinical manifestation of COVID-19 in children is generally more mild and less morbid than in adults [5]. Further correlation of COVID-19 in children with hepatic disease, and specifically biliary atresia, are not available. Our review demonstrated no documented cases of biliary atresia in a previously COVID-19 positive infant, though there are two cases of children developing hepatitis with COVID-19 requiring liver transplant [6].

Biliary atresia is a highly morbid disease leading to liver failure that is fatal if untreated. For children diagnosed early in the disease course, treatment involves creation of a hepatoportoenterostomy to reestablish bile flow, a procedure known as the Kasai repair. While advances have decreased the morbidity and mortality of this rare disease, liver transplantation offers the only hope for children later in the disease course or who fail a Kasai procedure [2]. Existing literature suggests about half of patients require liver transplant within two years of Kasai whereas over half survive years with the native liver, and some never progress to fulminant hepatic failure [7].

We report on a two-month-old female infant with direct hyperbilirubinemia who tested positive for COVID-19, was diagnosed with biliary atresia, and treated with a Kasai procedure. Potential associations between biliary atresia and COVID-19 infection are summarized. This case report was written within SCARE and PROCESS criteria and informed consent was obtained from the patient's guardian [8], [9].

## 2 Presentation of case

The patient is a two-month-old, term-gestation female, with a prior medical history notable only for failure to thrive during her first month of life and transient jaundice which resolved without intervention during week of life two. There was no prior surgical history and the baby was not taking any medications. There was no family history of biliary atresia, or any genetic disorders. At ten weeks of age, the mother sought care from the pediatrician when the patient's eyes appeared increasingly yellow. Laboratory testing demonstrated elevations in bilirubin (total 10.1 mg/dL, direct 7.9 mg/dL), alkaline phosphatase (1005 U/L), AST (247 U/L), and ALT (362 U/L). A nasopharyngeal swab for COVID-19 PCR collected for routine screening was positive. Infectious disease workup, including cytomegalovirus, was otherwise negative. In accordance with our hospital infection control policy, the patient was quarantined for two weeks. They did not develop symptoms associated with COVID-19, specifically no fever, cough, or other respiratory symptoms. Repeat labs two weeks later showed progressive elevations in alkaline phosphatase (1420 U/L), AST (292 U/L), and ALT (426 U/L). The patient's mother reported the infant had non-bloody, non-bilious, non-projectile emesis the proceeding day and was feeding poorly. On exam, the patient had scleral icterus and increased tone of the upper extremities. She was in no apparent distress and her stool was brown. The patient was admitted to our academic hospital for failure to thrive and dehydration, and she was considered to be recovered from a prior COVID-19 infection by evaluation of the infectious disease team.

**Fig. 1:**
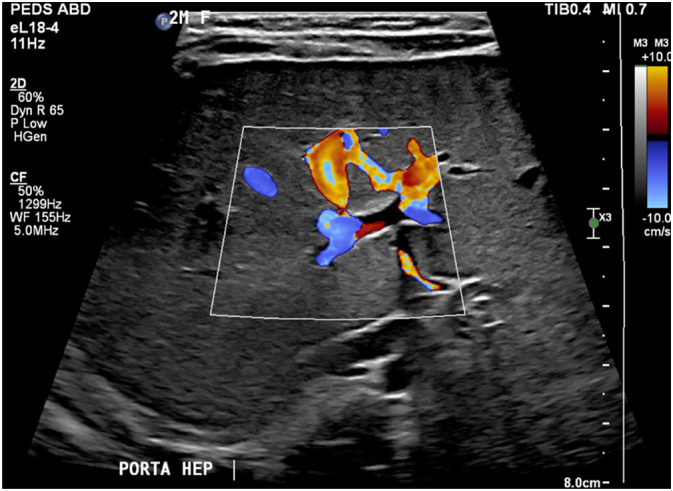
Ultrasound of liver demonstrating patent hepatic vasculature at the porta hepatis.

**Fig. 2:**
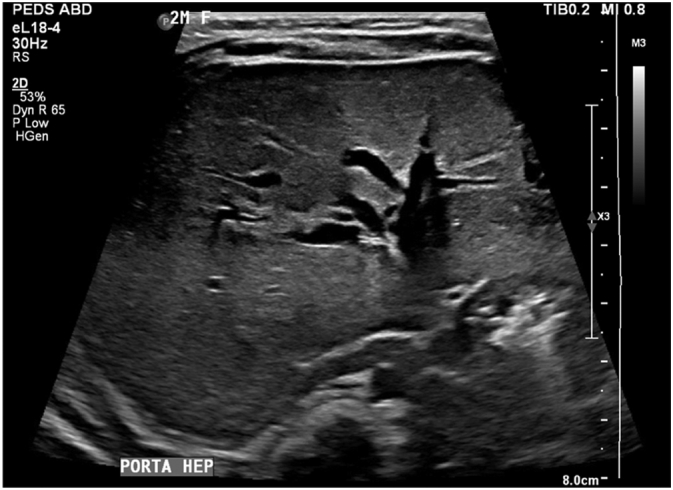
Ultrasound of liver at the porta hepatis showing complete absence of a gallbladder structure.

**Fig. 3:**
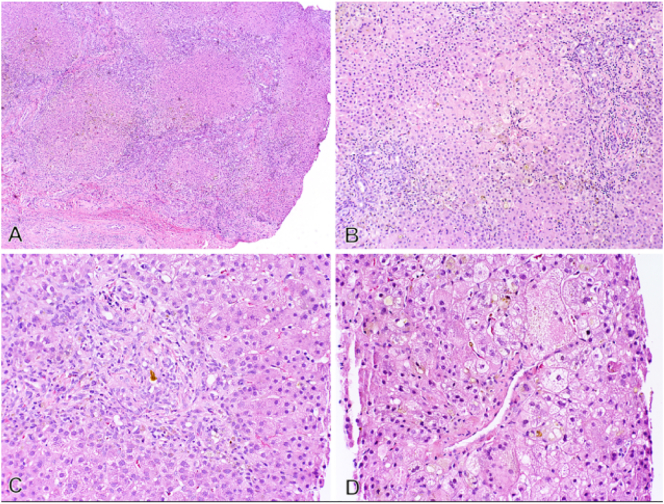
Representative photomicrographs of liver needle core biopsy (A&B) and excised portal plate from Kasai procedure (C&D). A) Portal tract with brisk bile ductular reaction and interlobular bile ducts with features of mild injury and a bile duct plug. (H&E stain 20×). B) Centrilobular zone with marked canalicular cholestasis and cholate stasis with focal giant-cell transformation. (H&E 20×). C) Portal plate with a ‘biliary pattern’ of portal to portal bridging fibrosis and early nodule formation (H&E stain 2×). D) Lower power view of severe canalicular hepatocellular cholestasis and marked bile ductular reaction (H&E stain 5×).

After admission, the patient underwent a liver ultrasound and percutaneous core liver biopsy. Ultrasound demonstrated a sonographically normal liver without nodularity or cirrhosis and patent hepatic vasculature (Fig. 1) but failed to visualize the gallbladder (Fig. 2). The biopsy demonstrated marked canalicular cholestasis, centrilobular cholestasis, mild interlobular bile duct injury with occasional bile duct plugs, and mild portal fibrous expansion without bile duct paucity (Fig. 3A & B). Overall, these findings were of a non-specific cholestatic pattern of injury, compatible with biliary atresia. Given the clinical, radiologic, and histologic findings, the pediatric surgery service was consulted and at twelve weeks of age the patient urgently received an exploratory laparotomy with planned cholangiogram for suspected biliary atresia. As is our institution's practice, the operation was performed collaboratively by a pediatric and liver transplant surgeon.

Intraoperative evaluation demonstrated a sclerotic gallbladder. A cholangiogram resulted in contrast extravasation into the surrounding tissue. These findings were interpreted as consistent with biliary atresia, and the team proceeded with a Kasai hepatoportoenterostomy. The sclerotic gallbladder and biliary structures were resected, with care taken to preserve the hepatic arteries and portal vein branches. A portoenterostomy was constructed using a Roux limb of jejunum as previously described [10].

The patient recovered well postoperatively without significant complication. The final pathology revealed extensive luminal obliteration of biliary structures as well as a cholestatic pattern of injury with bridging fibrosis and nodule formation (Fig. 3C & D). In the context of the operative findings, the histologic picture was consistent with the diagnosis of biliary atresia.

The patient has continued to do well, with her most recent follow-up at approximately one-year post-op. Her liver enzymes remain elevated but are overall improved and her bilirubin levels have normalized. There has been no icterus, jaundice, acholic stool, vomiting or abdominal distension. An ultrasound at four months post-op showed no abnormalities. She has had no apparent long term-sequelae of COVID-19 and has required no special considerations after the intervention.

## 3 Discussion

Biliary atresia is a heterogenous disease of unknown etiology, though multiple viral inflammatory factors have been suggested to play a role [11]. Implicated viruses include rotavirus, reovirus, herpes virus, Epstein Barr virus (EBV) and cytomegalovirus (CMV) [12], [13], [14]. A recent study demonstrated CMV DNA in half of infants with biliary atresia, with other studies demonstrating that over half of biliary atresia patients have a hepatic IgM and T-cell response to CMV [15]. Other work has shown a seasonal clustering of biliary atresia which provides support for the hypothesis that viral exposure may contribute to biliary damage [16]. Still, other studies have shown no such relationship between biliary atresia, viral exposure, and environmental factors [17].

COVID-19 is a novel virus associated with liver damage in some patients [4]. These patients usually present with elevated transaminases of unclear etiology early in their COVID-19 disease course. In patients who have died from COVID-19 and undergone autopsy, hepatic injury is relatively common, manifested histologically as hepatocellular microvascular steatosis [4]. One study found that abnormal liver tests occur in the majority of hospitalized patients with COVID-19 and suggested that such abnormalities may be associated with worse outcomes [18]. Other work has highlighted that liver injury is more common in patients with severe symptoms of COVID-19 and posits a role for CD4+ and CD8+ cells as mediators of hepatic insult [19]. Although the evidence for COVID-19 associated liver injury is increasing, the cause is unclear [19]. Evidence exists to support direct injury by the virus on liver cells, ischemic damage, drug induced insult, and exacerbation of prior liver injury [20].

We suggest that the liver inflammation associated with COVID-19 may similarly contribute to the development of biliary atresia in a neonate, but the shared rarity of pediatric COVID-19 and biliary atresia makes this a difficult hypothesis to test. Available data suggests that children are more likely to have a mild case of COVID-19 and are less likely to develop liver injury compared to adults. Authors have hypothesized that cases leading to liver injury in infants may result from use of antiviral drugs, rather than direct viral injury [20].

The only case reports of biliary atresia in the setting of COVID-19 describe patients who received liver transplantation after a failed Kasai repair, a scenario different from the one reported here [6], [14]. The timeline of these reports places the COVID-19 diagnosis after the biliary atresia diagnosis, whereas in our patient, diagnosis of COVID-19 came before the development of biliary atresia. Cirrhosis is commonly identified in patients with biliary atresia at the time of Kasai, which is usually performed within the first 90 days after birth. Therefore, the short time between COVID-19 diagnosis and intervention does not preclude a possible association. In presenting the first report of a patient with biliary atresia who was previously diagnosed with COVID-19, we raise the possibility that COVID-19 may have contributed to this neonate's biliary injury and suggest that addition study is needed.

## 4 Conclusion

Biliary atresia is a disease of potentially mixed etiology. Extrahepatic biliary inflammation resulting from a viral trigger is one factor thought to drive bile duct destruction. This, however, is a debated topic and no consensus has been reached. The COVID-19 virus causes a systemic inflammatory response and has shown tropism for the liver. This case presents the first reported occurrence of biliary atresia in an infant with COVID-19 and raises the question of whether COVID-19 could have contributed to the development of biliary atresia. More evidence is needed to understand the connection between viral exposure and biliary atresia. Additional investigation is necessary to understand if a relationship exists between COVID-19 and neonatal hepatic injury.

Written informed consent was obtained from the patient for publication of this case report and accompanying images. A copy of the written consent is available for review by the Editor-in-Chief of this journal on request.

## Provenance and peer review

Not commissioned, externally peer-reviewed.

## Sources of funding

None.

## Ethical approval

In accordance with Duke IRB protocol.

## Consent

written consent obtained and readily available.

## Research registration (for case reports detailing a new surgical technique or new equipment/technology)

Not applicable.

## Guarantor

Tamara Fitzgerald

## CRediT authorship contribution statement

Steven Thornton – chart review, writing.

Raluca Gosman – chart review, writing.

Debra Sudan – conceptualization, design, editing.

Henry Rice – conceptualization, design, editing.

Mitchell Arbogast – chart review, writing.

Tamara Fitzgerald – writing, editing, conceptualization, design.

## Declaration of competing interest

None.
